# *Isodon rubescens* research literature based on Web of Science database for visual analysis: A review

**DOI:** 10.1097/MD.0000000000041945

**Published:** 2025-05-02

**Authors:** Shaowei Xu, Jing Wu, Qingshan Yang, Huqiang Fang, Teng Xu, Bing He, Na Chen, Shihai Xing

**Affiliations:** a College of Pharmacy, Anhui University of Chinese Medicine, Hefei, China; b Institute of Traditional Chinese Medicine Resources Protection and Development, Anhui Academy of Chinese Medicine, Hefei, China; c Joint Research Center for Chinese Herbal Medicine of Anhui, Institute of Health and Medicine, Hefei Comprehensive National Science Center, Bozhou, China; d MOE-Anhui Joint Collaborative Innovation Center for Quality Improvement of Anhui Genuine Chinese Medicinal Materials, Hefei, China; e Anhui Province Key Laboratory of Research & Development of Chinese Medicine, Anhui University of Chinese Medicine, Hefei, China.

**Keywords:** bibliometrics, citespace, *Isodon rubescens*, the R programming language, VOSviwer

## Abstract

*Isodon rubescens* has been used as an herbal medicine in China for a long time. The significant value of development and utilization is affirmative. Bibliometrics is used as an approach to sort out, analyze, and visualize relevant literature in a particular field. So, it can intuitively express the research trends, hot directions, significant achievements, core journals, and outstanding authors in the field. But there is no bibliometrics analysis of *I rubescens*. The relevant dataset was retrieved and exported from the Web of Science database, and the results were obtained and visualized using the R Programming Language, CiteSpace, and VOSviewer, with the creation of time zone maps also using Scimago Graphica and Gephi. There were 506 valid data retrieved and 465 analyzed data selected. The country with the most significant number of publications is China, the institution with the largest annual publication volume is China Pharmaceutical University, the publication with the most relevant literature is the *International Journal of Oncology*, and the author with the most publications is Zhou. The keyword with the greatest intensity is “matastasis,” which is also an emerging keyword. The role of *I rubescens* has been continuously diversifying. It has been proven to play a role in treating major diseases such as multiple cancers, leukemia, liver and kidney function impairment, and cardiovascular and cerebrovascular diseases. This study will highlight the main research direction in this field, namely the use of *I rubescens* for the treatment of cancer.

## 1. Introduction

*Isodon rubescens* (Hemsley) H. Hara is used as traditional Chinese medicine. Doctor of traditional Chinese medicine claims it has the effect of clearing heat and detoxification, activating blood and relieving pain, and is commonly used in sore throats, fistulas and snake bites. After years of research, *I rubescens* has been found to have a significant inhibitory effect on cancer,^[1]^ especially digestive tract cancer. In recent years, researchers have been exploring other effects, such as anti-inflammatory,^[2]^ antibacterial,^[3]^ and lowering blood sugar^[4]^ in an attempt to find more clinical applications of *I rubescens*. Primary bioactive substances contained in *I rubescens* are terpenoids,^[3,5]^ such as oleanolic acid and ursolic acid. Most of them belong to the ent-kaurene diterpenoid. The earliest isolated compound, named oridonin, is a component of some modern clinical cancer treatment drugs. Studies have reported that oridonin is usually synthesized in the aerial part of the plant, and the relevant expression genes are concentrated in the leaves,^[6]^ which the content of oridonin is also similar to, including CYP796V^[7]^ and IrUGT86A1.^[8]^ In addition, *I rubescens* has been used as a tea beverage in China for a long time. Therefore, in some regions of China, it is cultivated as an economic crop.

Bibliometrics is a statistical method that integrates literature, which can digitize and visualize the commonalities, trends and connections of amount of literature,^[9]^ which helps researchers quickly understand the overall occurrence process, current latest directions and hot spots in a particular field. According to research, most disciplines want a high-quality review^[10]^ to solve the problem of reading a large amount of literature in search of a direction to explore. Traditional literature can do this, which can describe the current hot scientific research, but the connections and trends between various studies will not be concise and intuitive. This part can be expressed well by bibliometrics, using pictures such as co-existing network diagrams, surge words, time zone maps, timeline diagrams and other pictures, which can intuitively reflect the ongoing research.^[11]^ However, there are still many limitations in bibliometrics, such as the need for manual screening and condition setting during the process, which can have a certain subjective impact on the final results.

In this article, we used bibliometrics to analyze the literature of *I rubescens*, counted the countries, institutions, journals, authors and keywords of articles published in the field, drew tables and pictures, summarized and predicted research trends in this field, and provides a reference for *I rubescens* related research.

## 2. Data and method

### 2.1. Source of data

The data was sourced from the Web of Sience (WoS) database, with a total of 781 records. After many explorations, *Rabdosia rubescens*, *I rubescens*, Oridonin and pind were used as search terms, and the search time range was set from 2000 to 2023 (as of February 28, 2023).

### 2.2. Data filtering

#### 2.2.1. *Filter*

Inclusion criteria: Research literature on *I rubescens* in the field of medicine.

Exclusion criteria: duplicate studies; conferences, newspapers, reports, achievements, patents, information, and revoked publications; the theme is not related to traditional Chinese medicine; literature with incomplete information such as author, keywords, journal name, and publication time.

The retrieved questions are imported into the Note Express and EndNote literature managers for aggregation, and qualified documents are identified according to the above criteria to obtain qualified documents.

#### 2.2.2. *Data visualization*

The qualified documents screened by Note Express and EndNote are saved in a recognizable format of CiteSpace (version 6.2.2)^[12]^ and VOSviewer (version 1.6.19),^[13]^ imported into the software for analysis, and the output file after document processing is obtained. Import it into R for secondary analysis, and visualize the results with Scimago Graphica (version 1.0.34)^[14]^ and Gephi (version 0.10.1).^[15]^

CiteSpace parameter settings: time slicing is 2007 to 2023, time slicing is 1 year; G-index set the K value to 15; node type select institution, keyword; pruning chooses pathfinder and pruning sliced networks.

## 3. Results and analysis

### 3.1. Analysis of the annual distribution of literature publications

**Figure 1.**
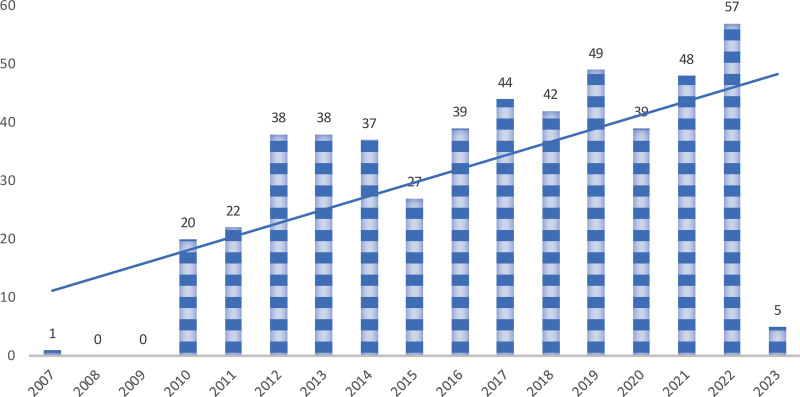
The number of document on *I rubescens* per years.

After searching and screening the WoS database, 506 valid data were obtained, excluding 6 early articles, 1 process article, 1 focus explanation article, 1 collaborative article, 1 explanatory article, 1 letter and 30 conference papers. After removing the data listed above, 465 analytical data were selected, including 448 articles and 17 review articles. In general, it can be divided into 2 periods, the first is the quiet period, which is a period when there are fewer researchers interested in this area and few articles are included. The first article was included in 2007 (Fig. 1), and there were no articles for 2 years after that. The second stage is the period of rapid growth, that is, from 2010 to 2023. Although there are fluctuations, the number of papers published in these 10 years has been rising, reaching a peak of 57 papers in 2022. From the trend point of view, this field will continue to be watched.

### 3.2. Analysis of documents issued by countries and institutions

**Figure 2.**
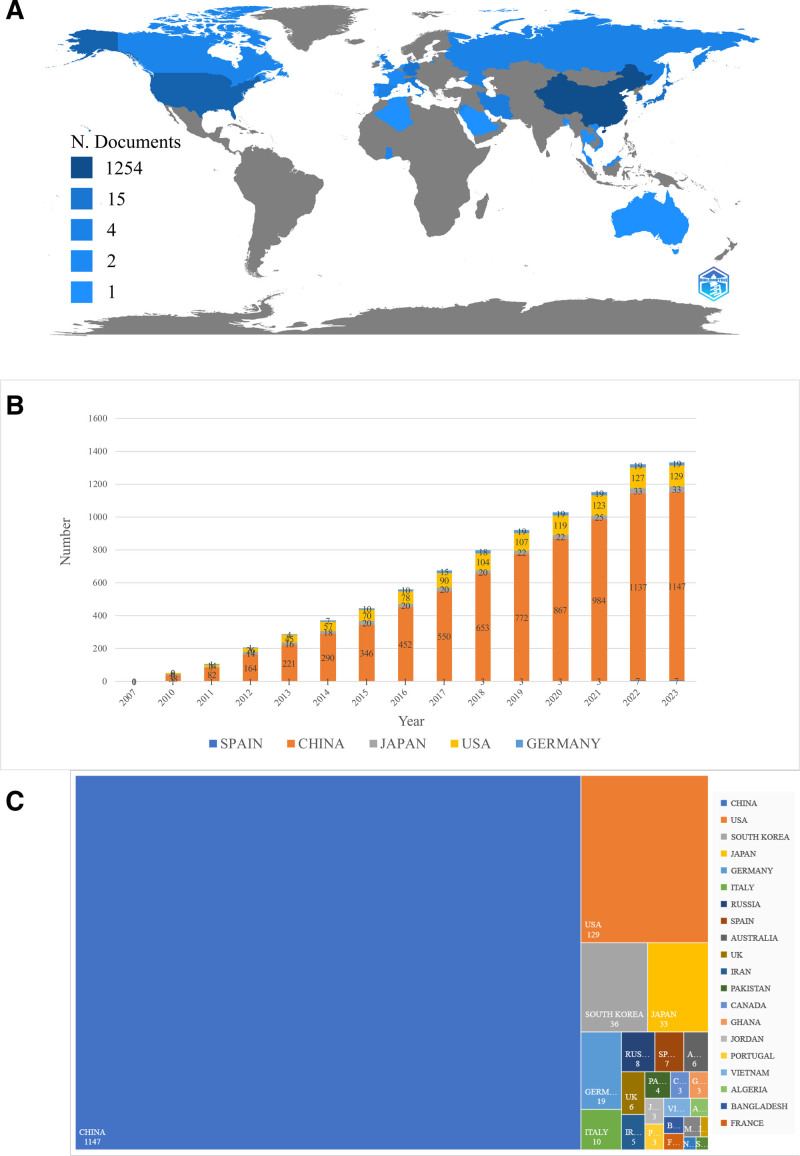
Analysis of country. (A) The map of county scientific production. (B) The number of publications per country per year. (C) The treemap of proportion per country’s publications.

According to the map obtained from the Wos literature data, the darker the color, the more posts are published. It can be seen that China and the United States are located in the darkest color, indicating a large number of articles in this field (Fig. 2A). As can be seen from Figure 2B, 26 countries have contributed to the literature in this area. China contributed the most papers (1147), accounting for 79.87% of the total publications, followed by the United States (129), South Korea (36), Japan (33) and Germany (19). The number of publications in the United States and South Korea is only equivalent to 10.61% and 2.87% of China, indicating that China paid more attention to the research and development of winter grass earlier. In Figure 2C, the annual publication volume of 5 countries is shown in different colors, China is the most prolific country, and the number is increasing year by year, and the United States publishes more than 100 articles per year.

**Figure 3.**
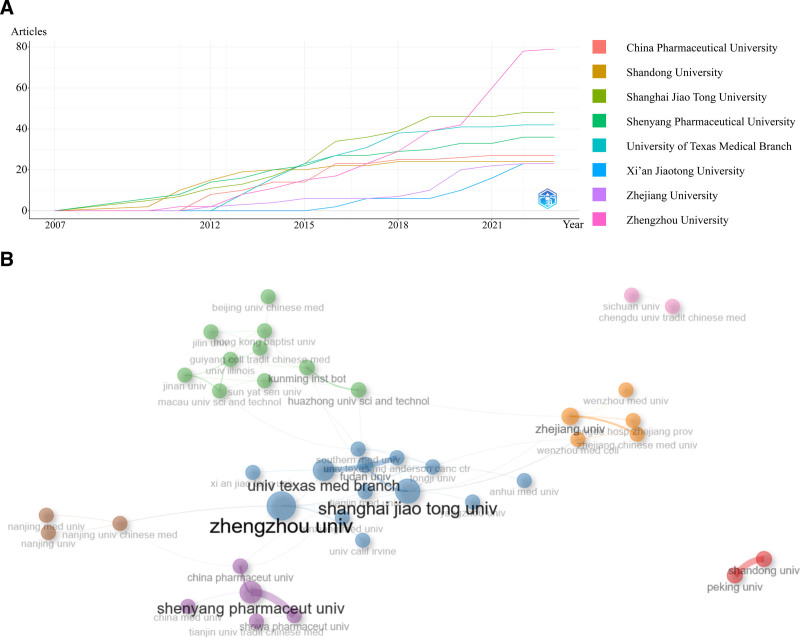
Analysis of affiliations. (A) The number of affiliation’ production over time. (B) The collaboration network of institutions.

According to statistics, 472 institutions have contributed to research in this field. Figure 3A depicts the annual publication volume of the top 8 institutions, with China Pharmaceutical University (China Pharmaceut univ) being the institution with the fastest annual production growth, and Shanghai Jiao Tong University (ShangHai JiaoTong University) maintaining the highest annual publication volume until 2021, indicating that the research focus of the institution is shifting. Six of the 7 institutions in this figure belong to China, indicating that China has made an important contribution to the research of winter grass. The Figure 3B is an institutional co-discovery network diagram generated by the R language. The size of the circle reflects the number of research done by the institution, and the thicker the connection, the closer the cooperation. With Zhengzhou University, Shenyang Pharmaceutical University and Zhejiang University as the center, the institutional cooperation network diagram on the research of winter grass was constructed, indicating that these institutions attach importance to mutual exchanges and cooperation while focusing on research in this field.

### 3.3. Publication analysis

Bradford law, also known as the law of documentary dispersion, was proposed by Bradford in 1948 and has been continuously improved. According to this theorem, journals can be divided into core journals, related journals, and unrelated journals. The journals covered in this article also roughly follow Bradford law (1:2.5:2.52), with an approximate number of 168 articles per division (Table 1). Therefore, a total of 26 journals were selected for analysis (Table 2). The number of applications can reflect the quality of the journal, and lists the articles’ total citations, citations per article and 2023 impact factor (IF) of the 26 selected journals. The largest number of publications was the International Journal of Oncology, with 13 articles, and the total number of citations also ranked first, indicating that the journal has a high acceptance rate for research related to winter grass. This is followed by International Immunopharmacology, Molecules and Oncology letters, which are also the main forums in the field. Cell Death & Disease is of high quality, as evidenced by the most cited data per article, suggesting that research on *I rubescens* is more focused on its therapeutic effects and mechanisms for cancer, and the journal with the highest IF is Cancer Research.

**Table 1:** The analysis of journals based on Bradford law.

Zone	Publication	Number of journals	Number of document
First zone	>4	26	166
Second zone	2 to 4	63	168
Third zone	1 to 2	163	172

**Table 2:** The number, reference, average citation per article and IF of journals.

Sources	Articles	TC	Average citations per articles	IF
International Journal of Oncology	13	312	24.00	5.884
International Immunopharmacology	11	173	15.73	5.714
Molecules	11	229	20.82	4.927
Oncology Letters	10	158	15.80	3.111
Molecular Medicine Reports	8	93	11.63	3.423
Acta Pharmacologica Sinica	7	165	23.57	7.169
Cancer Research	7	64	9.14	13.312
PLOS ONE	7	149	21.29	3.752
European Journal of Pharmacology	6	105	17.50	5.195
International Journal oPharmaceutics	6	219	36.50	6.510
Journal of Natural Products	6	157	26.17	4.803
Pharmaceutical Biology	6	68	11.33	3.889
Phytotherapy Research	6	37	6.17	6.388
Biochemical and Biophysical Research Communications	5	59	11.80	3.322
Biological and Pharmaceutical Bulletin	5	64	12.80	2.264
Biomedicine and Pharmacotherapy	5	129	25.80	7.419
European Journal of Medicinal Chemistry	5	222	44.40	7.088
Evidence-Based Complementary and Alternative Medicine	5	31	6.20	2.650
Frontiers in Pharmacology	5	46	9.20	5.988
International Journal of Molecular Sciences	5	91	18.20	6.208
Journal of Cellular and Molecular Medicine	5	79	15.80	5.295
Oncology Reports	5	161	32.20	4.136
Scientific Reports	5	86	17.20	4.996
Anticancer Drugs	4	56	14.00	2.389
Cell Death and Disease	4	193	48.25	9.685
Drug Development Research	4	34	8.50	5.004
Experimental and Therapeutic Medicine	4	45	11.25	2.751
Fitoterapia	4	57	14.25	3.204
International Journal of Molecular Medicine	4	91	22.75	5.314
International Journal of Nanomedicine	4	87	21.75	7.033

Abbreviations: IF = impact factor, TC = total citations.

### 3.4. Author information analysis

**Figure 4.**
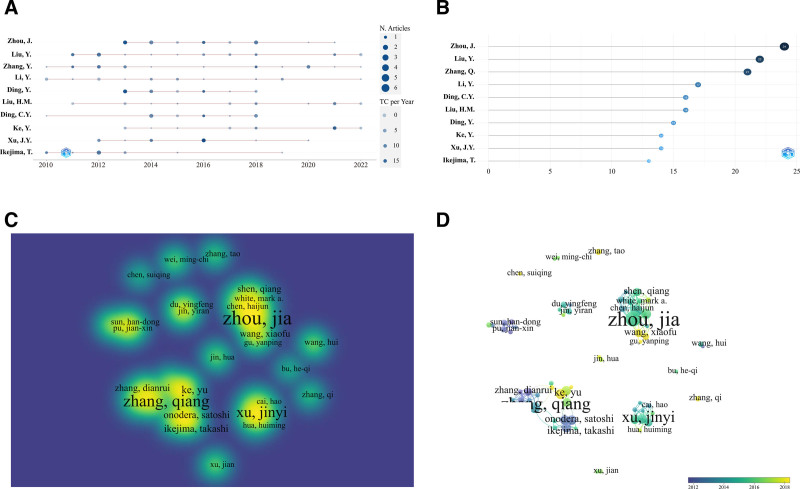
Analysis of authors. (A) The number of author’s production over time. (B) The top 10 number of author’s productions. (C) The overlay visualization of author’s collaborative network. (D) The density visualization of author’s collaborative network.

The number of publications and citations represent the author’s productivity and quality in the field, respectively. Visualize the number of articles published by authors per year (Fig. 4A), the color shade represents the citation, and the circle size indicates the number of posts. Among them, Zhou, Liu and Zhang are at the forefront of annual publications and citations. Figure 4B lists the top 10 researchers with enormous of articles, of which Zhou, J. published the most articles (24), followed by Liu, Y (22) and Zhang, Q. (21). Figure 4C is the fluorescence view of the authors, Zhou, Ding, Xu, Ke, Zheng, Onodera and Satoshi. The cooperative network has an intense fluorescence brightness, indicating that these groups have made outstanding contributions to the research of winter grass. Through VOSviewer visualize the cooperation between authors, the corresponding overlay collaborative network (Fig. 4D) is drawn, the color shade represents the time, the size of the circle represents the number of authors’ posts, and the connection represents the cooperation between researchers. Zhang and others have more research results, but the time has been relatively old, and the reference value may decline. Zhou, Chun and other authors have more connections between them, reflecting the importance of collaboration. Wang, Gu, Ke and other lightest colors are emerging authors in recent years, with larger circles and more connections, indicating that these researchers may continue to pay attention to the research field of winter grass.

### 3.5. Keyword trend analysis

**Figure 5.**
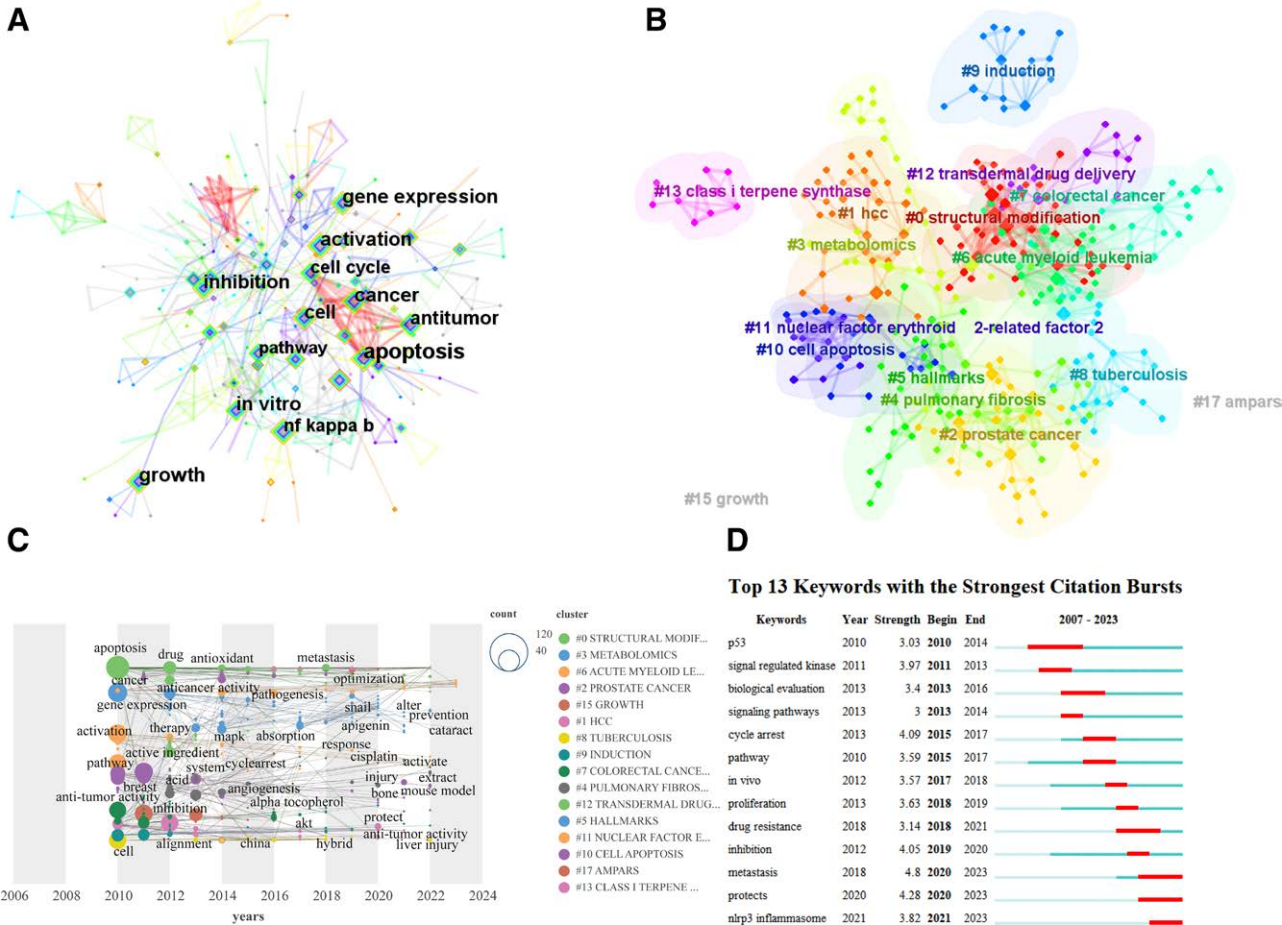
Analysis of keywords. (A) The co-occurrence network of keywords. (B) The cluster of keywords. (C) The time zone map of keywords. (D) Keywords with the strongest citation bursts.

Generally speaking, keywords are the center and main content of an article, so analyzing and sorting out keywords helps to grasp the overall trend and trend of field research. Using the CiteSpace algorithm to analyze the keywords, remove the keywords used in the search, and merge synonyms, the obtained data roughly conforms to Zipf law (constant is about 350). The corresponding co-occurrence network diagram (Fig. 5A) was obtained from the visual data, the higher the frequency of occurrence, the larger the square, the color represents the time, and the connection represents the simultaneous occurrence of keywords. Among all keywords, “apoptosis” appeared the most frequently, reaching 103 times, and the effect of winter grass on apoptosis was the focus of research in this area, followed by “activation” (68 times), “in vitro” (65), “expression” (62), “growth” (62), “cell” (57), It is indicated that the influence of Dongling grass on cell activity through in vitro experiments may be a hot direction in research. The keyword cluster plot shows 17 categories (Fig. 5B), which can be classified into 2 categories: the therapeutic mechanism of the disease and the innovative production of the agent, and it is possible that the research on the winter grass focuses on the mechanistic impact on cancer. CiteSpace, Gephi, and Scimago Graphics combined to make a time zone map of keywords (Fig. 5C), the legend position on the right corresponds to the location of the cluster in the figure, the size of the circle represents the frequency, and the abscissa is the year of its occurrence. “Aproptosis,” “activation,” and “antitumor” are the first batch of keywords that appear, influencing the clustering of words that appear later. “cataract” and “accuracy” are the latest to appear in 2023, due to the short time, their frequency is not high, but it may continue to appear in the future, indicating that ophthalmic medical treatment and precision drug delivery may be the research hotspot of *I rubescens* in the future. Finally, a schematic diagram of emergent words (Fig. 5D) was drawn, and a total of 13 keywords with an intensity of 3 or more were selected and sorted by the start year of emergence. “P35” is the earliest word to appear, with a strength of 3.03. The intensity of “metastasis” was the highest (4.8), indicating that there is more literature related to cell metastasis, and the metastasis of cancer cells has always attracted the attention of medical institutions, indicating that the research of *I rubescens* is closely related to cancer.

## 4. Discussion

The 46 records were singled out as the basis for the analysis, and the number of publications per year showed an upward trend, suggesting that the research of *I rubescens* is likely to continue to emerge. In the world map and the number of national publications, China obviously occupies a large proportion. The United States, the second largest in number, has stabilized at more than 100 articles per year. China has the highest number of publications, which may be related to the fact that *I rubescens* is an economic crop in some regions of China. Not only as a source of traditional Chinese medicine, but also as a raw material for chemical drugs, as well as a tea beverage for daily consumption.

The research directions of the top 5 institutions published each year have their own characteristics, and the literature included in China Pharmaceutical University is basically related to cancer, and the synthesis and construction of derivatives of Oridonin,^[16–23]^ and explains the mechanism of apoptosis in cancer cells.^[24,25]^ China Pharmaceutical University and Shandong University jointly proposed the application of fluorescent probe technology in the mechanism.^[26]^ They found that Oridonin usually accumulates in the mitochondria of cells in large quantities, which provides a reference for subsequent research. Shandong University focuses on the construction of nanocarriers carrying Oridonin,^[27–30]^ including nanogels,^[31]^ nanoparticles,^[27,32–34]^ nanosuspensions^[35–38]^ and nanocrystals.^[39]^ The purpose of the study is mainly for clinically targeted drug delivery, and there is usually a related collaboration with Peking University,^[31,32,39,40]^ of course, the mechanism of ovarian cancer^[41]^ and prostate cancer^[35–37]^ being affected by Oridonin. The research at Shanghai Jiao Tong University can be divided into 2 directions, 1 focusing on the efficacy of *I rubescens* in leukemia,^[42–45]^ and the other is the combination of drugs,^[46]^ which crosses.^[43,44]^ Other aspects of research are more complex, involving mostly the mechanism of the action of *I. rubescen* on cancer, such as the combination of certain drugs to treat tumors, which can enhance each other’s efficacy.^[46]^ Shenyang Pharmaceutical University, ranked third, focuses on the mechanism of apoptosis and phagocytosis,^[47–49]^ and currently the pathways or components studied by the institution include JNK,^[50]^ NO-ERK-p53,^[51]^ NF-Kb-COX-2-IL-1B,^[52]^ and c-Met^[53]^ to illustrate the potential mechanism of inhibition of various cancer cells by *I. rubescen*. Among them, it is worth noting that recent research combining multiband blending analysis, electrochemical oscillation pattern, and oxidation resistance analysis to detect the quality of the tablet named Donglingcao.^[54]^ It may be precise and accurate, but the simplicity is also reduced, exploring the most iconic detection component of *I. rubescen* can be the key to quality control. The University of Texas Medical School differs from the previous 4 universities in that the literature focuses on the construction of derivatives^[55,56]^ and the study of inflammatory mechanisms.^[57–59]^ The University of Texas Medical School has developed a variety of oridonin analogs^[60]^ and synthetic methods,^[61]^ which have become a potential alternative to oridonin resistance,^[61]^ targeting mainly hepatitis^[62,63]^ and enteritis.^[59]^ In recent years, there have been more studies on the possibility of liver fibrosis^[62–65]^ and intestinal fibrosis^[66]^ protected and cured by oridonin. The collaboration between China Pharmaceutical University and Shenyang Pharmaceutical University is the construction and design of derivatives,^[19–22,25,67]^ mostly anti-inflammatory and antibacterial drug candidates. The 2 universities are closely linked and have a long cooperation time span, and further cooperation may be carried out in the direction of the construction of oridonin derivatives. Zhengzhou University, Shanghai Jiao Tong University, and the University of Texas Medical School have collaborated with each other, but they are not very closely linked, and their research focuses on their respective directions, such as the mechanism of apoptosis and phagocytosis,^[68]^ the construction of oridonin derivatives,^[69]^ and the possibility of treating hepatitis.^[62]^

Three journals were selected from the source, with the most publications, the most citations and the most IFs. The journal with the most articles is the International Journal of Oncology, in which most of the articles are related to combination drugs (38.46%), and Oridonin can enhance the effects of drugs such as arsenic trioxide,^[70]^ NVP-BEZ235,^[46]^ gemcitabine^[71]^ and cecuximab.^[72]^ Interestingly, the MAPK (mitogen-activated protein kinase) pathway has been mentioned several times in related articles,^[71,73–75]^ suggesting that MAPK is an important pathway for Oridonin to inhibit cancer cells or induce apoptosis. The International Journal of Oncology suggests that the journal prefers the combination of drugs, which may be one of the future research directions of *I. rubescen*. Cancer research (IF = 13.312), the journal with the highest IF in the table, in which the literature on *I rubescens* deals more with derivatives and analogs for the treatment of breast cancer,^[56,76–78]^ suggesting that the journal may be more focused on the research that can be transferred to actual production and life-impacting research. Cell death and disease (44.40 points) with the highest citation score for each article in the table, and exploring the mechanism by which *I rubescens* induces apoptosis and phagocytosis to achieve anticancer effects is the main literature category in the journal.^[79,80]^ It is interesting to note that Oridonin is proposed as an alternative to drug resistance, and the above 3 journals have articles on,^[81–83]^ comparing Oridonin with other drugs to form a substitute may also be a future research direction.

The author, Zhou, was the most prolific, participating in the completion of 24 papers, including 10 conference papers, 1 review, 13 research articles, 2 signed as first authors,^[82,84]^ and 2 articles with the highest IFs reached 9.76 points^[69]^ and 9.69 points^[82]^ respectively. His earlier articles focused on the modification of the A-ring of Oridonin and the synthesis of corresponding analogues,^[55,61,85,86]^ the enhancement of A-ring aaidatinn, its water solubility and bioavailability. His medium-term delve into the study of hepatitis and liver fibrosis.^[62,63]^ Recent publications have dealt with D-ring modification and the synthesis of analogues,^[69,87]^ and are more specific to breast cancer. Interestingly, the creation of a D-ring modified analogue by aziridination was first proposed by his team,^[69]^ which is also the article with the highest IF, and another first-author high-scoring article published earlier also involved the synthesis of analogues,^[82]^ which could be a potential alternative to kidney cancer drug resistance. The authors also led a team to study the role of Oridonin on angiogenesis,^[84]^ hoping to find a way to reverse cancer cells and normalize blood vessels in cancer cells. Ikejima, T. has 13 research articles, all focusing on the effects of other substances on the reaction process of Oridonin in the body. Studies have shown that ROS (reactive oxygen species) promotes apoptosis in cells, while Oridonin induces its production,^[49]^ while hydroxyl radical,^[88]^ tyrosine^[50]^ and NO^[51]^ promote Oridonin apoptosis-induced effect. However, components including EGFR (epidermal growth factor receptor),^[89]^ HGF (hepatocyte growth factor)^[52]^ and c-Met^[53]^ will play the opposite role. It is worth mentioning that NO has an impact by forming a positive cycle of NO-ERK-p53^[51]^ and regulating NF-Κb-COS-2-IL-1β.^[90]^ The article with the highest IF suggests that inhibition of EGFR can enhance the effect of Oridonin,^[89]^ and that combining EGFR-targeted drugs with Oridonin may be a practical anticancer approach. The author with the highest H-dex score is Liu, with 22 articles, all of which are research literature. It involves diseases such as cancer, leukemia^[91,92]^ and high-oxygen lung damage,^[93]^ focusing on pharmaceutical agents’ innovation and practical application. In order to improve the pharmacokinetics of oridonin in vivo, components including galactose,^[31]^ MCF-7 (Michigan cancer foundation-7),^[38]^ GAS (gas anti-solvent),^[94]^ PEG-NLC (polyethylene glycol-Modified nanostructured lipid carriers),^[40]^ PLA-RGD-NPs (ORI-loaded atactic poly(D,L-lactic acid) nanoparticles),^[95]^ NLC (nanostructured lipid carriers),^[29]^ GC-NP (nanoparticles coated with galactosylated chitosan),^[32]^ ADC (antibody-drug conjugates)^[96]^ and other components are used to carry oridonin to increase its retention time. Different kinds of derivatives have also been constructed,^[97,98]^ expanding the scope of oridonin or as an alternative to drug-resistant diseases.^[81,99]^ Liu, Y. mentioned iron death in his article with the highest impact factor (IF = 14.90),^[100]^ which has only 3 articles in the research field of *I. rubescent*. And all of them are high-scoring works in the past 2 years, indicating that the study of the mechanism of action of oridonin inducing iron death to achieve anticancer may be one of the hot spots in the future.

Keywords are an important component that reflects the main idea of articles. Keywords can be used to understand the field of its research direction. From Figure 5B, it can be seen that keywords are divided into 2 categories: the production and innovation of drugs and the mechanism of action on diseases. The first type is the mechanism of action on diseases, most of which involve cancer, including test tube squamous cell carcinoma,^[101]^ laryngeal cancer,^[102]^ gastric cancer,^[103]^ colon cancer,^[84]^ breast cancer,^[104]^ pancreatic cancer,^[105]^ and lung cancer.^[106]^ In addition, it also extensively involves major diseases such as depression,^[107]^ enteritis,^[108]^ cardiovascular and cerebrovascular diseases,^[109–111]^ liver injury,^[112]^ renal failure^[113]^ and leukemia.^[96]^ The above results indicate that *I rubescens* can play a certain role in various diseases, especially digestive tract diseases. However, it also indicates that *I rubescens* has not been found to significantly affect in a certain disease, so further exploration is needed. The second category is the innovation and production of medicaments. Researchers have used materials and methods, such as nano preparations^[39]^ and liposomes^[114]^ to carry the effective substances in *I rubescens* such as Oridonin, so that they can stay in the human body for a longer time to enhance the efficacy. In recent years, articles have focused on the combined use of drugs, allowing Oridonin to have synergistic effects with other drugs,^[115]^ while also partially reversing the tolerance to drugs.^[116]^

Studies have shown that *I rubescens* has a therapeutic effect on esophageal squamous cell carcinoma by inhibiting the protein expression of LASP1 (LIM and SH3 protein 1) and PDLIM1 (recombinant PDZ And LIM domain protein 1),^[101]^ and selectively producing cytotoxicity on p35 mutant cells.^[117]^ Interestingly, in the concomitant drugs for this cancer, Getuximab enhances Oridonin ‘s induction of cell apoptosis,^[118]^ while Oridonin can enhance the effect of cisplatin.^[117]^ These 3 drugs may be used in combination. Oridonin inhibits Wnt/ β- Chain protein signaling pathway,^[119]^ TGF- β The 1/Smads PAI-1 signaling pathway,^[120]^ colon cancer cell metastasis^[121]^ and JAK2/STAT3 signaling pathway^[84]^ to resist colorectal cancer. It is worth noting that Oridonin can enhance MAPK activation by upregulating BMP7 (bone morphogenetic protein 7)^[122]^ and reduce PTEN (phosphatase and tensin homologue) phosphorylation^[123]^ to achieve therapeutic goals, as AMPK/mTOR/ULK1 can induce apoptosis in colorectal cancer cells.^[124]^ Interestingly, Oridonin upregulation of BMP7, p38, and P35 elements will enhance Oridonin induction effect,^[75]^ achieving a positive cycle. The process of Oridonin treating gastric cancer involves Apaf-1 (apoptotic protease activating factor-1),^[125]^ Caspase-3^[125]^ cytochrome c,^[125]^ CdK1 (cyclin-dependent kinases 1),^[126]^ and cyclin β,^[126]^ c-Met,^[127]^ mitochondrial signal,^[128]^ caspase-3,^[129]^ VEGF (vascular endothelial growth factor),^[130]^ integrin β,^[130]^ PCNA (proliferating cell nuclear antigen)^[130]^ and other pathways and elements. Oridonin promotes the apoptosis of cancer cells by enhancing the functional expression of p35,^[131]^ activating the phosphorylated JNK/C-JUN pathway,^[132]^ triggering the stop of the G2/M cell cycle^[133]^ and inducing the occurrence of the iron death mechanism.^[100]^ Studies have shown that Oridonin can reduce the levels of P-gp (P-glycoprotein), MRP1 (multi-drug resistance-associated protein), cyclin D1 and cancer inhibitor of protein phosphatase 2A in vivo, thereby reversing drug resistance.^[99]^ Recent articles have predicted the key targets of Oridonin on gastric cancer,^[103]^ which may be a reference direction for future research. In addition to severe gastrointestinal diseases, the number of reports related to leukemia is large, involving multiple regulatory methods. It is worth noting that different drugs are often combined in the treatment regimen. Oridonin collaborates with ATRA (all-trans-retinoic acid) to induce differentiation of NB4 and NB4-R1,^[134]^ inhibits LYN/mTOR through synergistic imatinib,^[135]^ regulates MAPK signaling through synergistic VPA (Valproic acid)^[73]^ and downregulates Bcl-2/Bax ratio,^[136]^ and regulates PBK/Akt/mTOR signaling through synergistic NVP-BEZ235.^[46]^ The mechanism of Oridonin reversing drug resistance has also been explained.^[81,83]^

In summary, based on the keywords and research trends in the paper, it can be seen that *I rubescens* has received much attention in the application of cancer, indicating that it does have a significant effect in this area. Studying in this direction may lead to the discovery of more cancer treatment methods. However, precisely because of this, the research direction is relatively single, and other aspects such as biological control, fertilizers, environmental beautification, and food have not been reflected. Perhaps *I rubescens* still has potential application value in these aspects.

## 5. Limitation

There are still several limitations in the current research. First, the data in this paper are all from the Wos, while other databases are not involved. The keywords used in this data retrieval are limited, thus other related words may be omitted. Moreover, reviews, reports, and conference papers were not analyzed. Some fundamental and milestone research that performs poorly after visualization may be overlooked. Due to the limited number of articles on some emerging hot topics, they may be regarded as unimportant parts. Finally, but importantly, bibliometric research is only a superficial and systematic field investigation and cannot cover all aspects. Of course, through this method, it can provide certain references for scholars in this field.

## 6. Conclusion

Through bibliometric analysis, past research about *I rubescens* is integrated and organized, then visually presented by visualizing. The number of studies related to *I rubescens* is showing an increasing trend year by year, indicating that this field has great potential and that a large number of studies will emerge in the future. Most of the institutions that contribute articles are in China, but the cooperation between Chinese researchers and other countries is far away. International Immunopharmacology, Cell Death & Disease and Cancer Research are closely related to cancer. It can be predicted that cancer treatment is the leading research direction of *I rubescens*, which will involve its mechanism and the improvement of dosage. However, it cannot be denied that further validation is needed to confirm the safety and effectiveness of using *I rubescens* as a drug.

## Author contributions

**Conceptualization:** Jing Wu.

**Data curation:** Shaowei Xu, Teng Xu.

**Formal analysis:** Qingshan Yang, Huqiang Fang.

**Funding acquisition:** Qingshan Yang.

**Investigation:** Shihai Xing.

**Methodology:** Shihai Xing, Shaowei Xu, Qingshan Yang, Huqiang Fang, Bing He.

**Project administration:** Teng Xu.

**Resources:** Jing Wu.

**Software:** Shaowei Xu.

**Supervision:** Bing He.

**Validation:** Na Chen.

**Visualization:** Shaowei Xu.

**Writing – original draft:** Shaowei Xu, Jing Wu.

**Writing – review & editing:** Shihai Xing, Qingshan Yang, Na Chen.
